# Fairness and randomness in decision-making: the case of decision thresholds

**DOI:** 10.1007/s11229-025-05091-7

**Published:** 2025-06-18

**Authors:** Kate Vredenburgh

**Affiliations:** Department of Philosophy, Logic, and Scientific Method, LSE, London, England

**Keywords:** Fairness, Randomness, Algorithmic decision-making, AI

## Abstract

This paper defends the role of lotteries in fair decision-making. It does so by targeting the use of decision thresholds to convert algorithmic predictions and classifications into decisions. Using an account of fairness from John Broome, the paper argues that decision thresholds are sometimes unfair, and that lotteries would be a fairer allocation method. It closes by dealing with two objections. First, it deals with the objection that lotteries should only be used to break ties in cases where individuals’ claims are equally strong. Here, the paper gives a new argument for Broome’s view, targeting decision criteria that are arbitrary and highly standardized. It then defends the arguments of the paper against the objection that lotteries are not morally superior to other methods of arbitrary choosing.

## Introduction

Very rarely do we encounter a perfect predictor. Predictions can fail to be accurate in different senses. Predictions may be biased, in the statistical sense: on average, a predictor may predict a different value for the population than the true value. Or, predictions may exhibit a high degree of variance: when a model is given the same input, it returns a different output. And, both bias and variance can create moral troubles when we use predictive models to inform the allocation of important social goods. If a hiring model is highly biased, it may suggest unqualified candidates for jobs, producing productive inefficiencies or harms from incompetence. If the same model also exhibits high variance, it may violate requirements of procedural fairness: two candidates that have the same qualifications will get different predictions as to how qualified they are for the job.

Accuracy has therefore received much of the focus in moral analyses of how to embed predictive models into human decision-making processes. One prominent example of the accuracy approach to fairness, as we might call it, is the field of algorithmic fairness. Research in computer science into algorithmic fairness began from a concern about observed differences in the distribution of error rates across different populations (Barocas et al., 2019: Chap. 3). One prominent example is ProPublica’s research into an algorithm to predict recidivism, or the likelihood of committing a crime after release from incarceration, which is used in the United States criminal justice system to inform decisions about bail and sentencing. Based on data they gathered on actual rates of re-arrest among White and African American individuals released from prison, ProPublica alleged that the algorithm was racially biased because it had a higher proportion of false positives for African American individuals. The company that developed the algorithm, COMPAS, alleged that the algorithm was not biased, because it satisfied another measure of accuracy, calibration, as the algorithm’s scores have equal evidential value at the population level across both groups. Philosophers have entered the debate over fairness metrics as well, defending one of these fairness criteria as necessary for a fair decision process (Hedden, 2021; Long, 2021), or arguing that none are (Eva, 2022).

Here, though, I want to home in on another aspect of algorithmic decision-making which creates concerns of unfairness: the use of decision thresholds to allocate goods and opportunities. A decision threshold is a function from a prediction, either continuous or discrete, to a decision. They seem to embody a desirable kind of consistency, contributing to the fairness of a decision (Mayson, 2019). But, as I will argue below, they can actually lead to unfairness, a point which has been overlooked.

To make this argument, I will draw on a theory of fairness from Broome (1984; 1990–1991) to further develop a Broomean account of fairness, according to which fairness is a matter of respecting claims in proportion to their strengths (Sect. 2). In Sect. 3, I use the Broomean account of fairness to argue that decisions produced by the use of decision thresholds are sometimes unfair, and that lotteries would be a fairer allocation method. I also argue that a Broomean account of fairness can rationalize research programs in computer science that aim to introduce more diversity into machine learning systems. In Sects. 4 and 5, I respond to two important objections. In Sect. 4, I deal with the objection that lotteries should only be used to break ties in cases where individuals’ claims are equally strong. Here, I give a new argument for Broome’s view that goods should be allocated in proportion to the strength of claims, that a Broomean account of fairness best balances considerations of the equality of people with the importance of giving each their due in light of institutionally-backed legitimate expectations. In Sect. 5, I argue against the objection that lotteries are not morally superior to other methods of arbitrary choosing.

By using Broome’s account of fairness to theorize about algorithmic decision-making, we end up at the surprising conclusion that a common practice - the use of decision-thresholds for decision-making - is unfair.^1^ This article thus contributes to the growing literature on algorithmic monoculture and the moral value of diverse or random algorithmic decision-making (Creel & Hellman, 2022; Jain et al., 2023). It is also one of many structural critiques of approaches to algorithmic fairness, encouraging us to look beyond the properties of one-off decisions to people’s life trajectories over time and how those are shaped by background institutions (Jain et al., 2023; Kasirzadeh, 2022). And, on the other hand, theorizing about algorithmic decision-making also leads us to a new Broomean account of fairness. Contingent but morally significant features of modern AI and surveillance systems, such as the scale on which they operate, the homogeneity of their outcomes, and the interconnected decision-processes fed by surveillance systems and the low cost of information sharing, point us towards the overlooked moral complaint that it is unfair if people are shut out of valuable opportunities in institutions that are core to building their life plans. And, this complaint pushes us to refine Broome’s theory, giving a pluralistic account of fairness that is grounded both in the equality of persons and in their separateness.

## Fairness

There are two moral bedrocks at the heart of any account of fairness. One is the concept of equality. Equality plays two important roles in the account of fairness. Because everyone has the same fundamental moral status, they are entitled to make certain kinds of claims on others just in virtue of being a person (Scheffler, 2003). Furthermore, people ought to be treated the same if they are the same in all the morally relevant respects that go beyond this fundamental status.^2^ If Sandra has two children of the same age, it is unfair if one can stay out all night while the other has a strict early curfew. The second moral bedrock at the heart of fairness is a notion of giving each their due (Cohen, 2008; Schouten, 2024). As Aristotle says, “[j]ustice […] should be equal for equal persons. But equality in what sort of things and inequality in what sort of things—this should not be overlooked.”^3^ Sandra may treat her children equally while failing to give each their due. Her children may protest that it’s unfair, for example, if all their peers have the freedom to see friends in the evening, but they do not.

The arguments of Sects. 3 and 4 will use John Broome’s account of fairness (1984; 1990–1991) to defend the claim that the use of decision thresholds in algorithmic decision-making is sometimes unfair, and lotteries would be a fairer allocation method. However, in order to do so, I will need not only to lay out Broome’s account of fairness, but also to develop it. That is because, as Piller (2017: 218) notes, not only is a theory of fairness not a complete theory of morality, “Broome’s theory is also incomplete as a theory of fairness.” Below, I will provide a richer account of claims, both their structure and their source, as well as an explanation of why the separateness of persons explains the structure of how claims combine and supports lotteries as a fair allocation procedure.

### The concept of fairness

Broome’s (1990–1991) account of fairness as *respecting claims in proportion to their strength* reflects both of these moral bedrocks. The idea of *respecting claims* cashes out what it is to give each their due. For Broome, claims are a certain type of reason. They are distinguished from other types of reasons in virtue of the fact that they generate directed duties, or duties owed to the particular person that is the bearer of the claim. Broome does not give a detailed account of claims, but he does say a few things to illustrate their nature that are important for what follows. Claim-based reasons are grounded in facts about need (or benefit more generally), promises or agreements, or desert. Promises or agreements are perhaps the clearest ground of claims, as they clearly generate directed duties. If Abid promises to mow his neighbor Beatrice’s lawn tomorrow because she has broken her leg, then he cannot discharge his duty to mow Chikako’s lawn instead. Much of the theorizing about fair allocation starts from intuitions about cases of equal and urgent needs, such as two individuals who need life-saving medicine. However, not all facts about need will give rise to a claim. To borrow an example from Piller (2017), the fact that I’ve run out of onions does not ground a claim that generates a duty for my neighbor to give me an onion. By contrast, if there has been a natural disaster, and I’ve run out of potable water, that fact would generate a directed duty for my neighbor to give me water, if they have a surplus. Furthermore, claims can be disjunctive, and they may be satisfied by a range of different goods or states of affairs. Consider the case of credit or paid employment. Both loans and the income and status from paid employment are necessary in modern societies in order to pursue one’s life plans, and to mitigate complaints from the unfair distribution of income and wealth.^4^ However, no creditworthy individual has a claim to a loan from any particular bank, nor does any qualified person have a claim to a particular job. Their claim is against a group of banks or employers; this structure of the claim is why I term it “disjunctive” (Dani has a claim that A offers them a job, or B does, or C does…). In addition, claims are often best described at a more abstract level, such as a claim to potable water in the case of a disaster, or life-saving medicine. But, the directed duty that the claim generates in a particular context will depend on features of the context. How much potable water someone is owed will depend on how many other people have a claim to it and how much potable water there is.

In addition, claims are also importantly distinct from reasons to bring about the best state of affairs. Broome motivates this distinction by example:

Someone has to be sent on a mission that is so dangerous she will probably be killed. The people available are similar in all respects, except that one has special talents that make her more likely than others to carry out the mission well (but no more likely to survive). This fact is recognized by her and everyone else. Who should be sent? Who should receive the good of being left behind? It could plausibly be thought that the right thing is simply to send the talented person. But it is also very plausible that doing so would be unfair to her, and that fairness requires a lottery to be held amongst all the candidates. (Broome, 1990–1991: 90)

The distinction between claims as directed duties and other types of reasons explains the intuition that there is a conflict between reasons of fairness and reasons of overall benefit generated by this example.^5^ There are reasons of aggregate welfare to send the most talented person on the mission, assuming that the act of successfully carrying out the mission creates more overall welfare than the act of failing to carry out the mission. Let’s assume that everyone has willingly joined a group that regularly undertakes dangerous missions. None of the group members has a greater claim than others not to be sent on the mission: in virtue of joining the organization and promising to undertake dangerous missions, they each acquire claims to be allocated such missions. In addition, claims are also a category of reasons that are different from rights-based reasons. In other words, claims are also not constraints, which are sufficient to require or rule out certain actions on their own. The right to private property, for example, rules out others from exercising control over someone’s property. For Broome, the plausibility of this distinction suggests that there is a distinct subset of reasons that are claims.

Broome, as well as others following him (e.g., Otsuka, 2012), take claims to be a special subclass of reasons that are unique in how they are treated in our deliberation. For reasons that are not claims, it is appropriate to weigh reasons against each other in order to determine the best action all things considered. Say that I’m deciding whether to drink my third coffee of the day. To do so, I weigh prudential reasons, such as the expense, against hedonistic reasons, such as the pleasure I take in its taste. But, according to Broome, claims ought not be weighed up across people in this way. It would be unfair if Amir’s greater claim to life saving medicine outweighed Aditi’s lesser claim. And that is because weighing up claims across people violates the separateness of persons, according to Broome.^6^ The separateness of persons is understood as a claim about the permissible balancing of benefits and burdens. While it is permissible to balance benefits and burdens across the life of a single person, it is not permissible to balance benefits and burdens across people. When it comes to claims, weighing claims across people violates a directed duty to someone if the good is allocated to some third party with a claim, even if that third party has a stronger claim. In other words, these directed duties can be neither satisfied nor silenced by allocating a good to another individual to whom a different duty is owed. The separateness of persons rules out weighing claims against each other.

The above line of reasoning, however, seems to land us in a vicious argumentative circularity. Claims are those reasons that ought not be weighed against each other in one’s reasoning. And, they ought not be weighed against each other because of the separateness of persons. However, the account of the separateness of persons above cashed it out as ruling out the aggregation of interpersonal reasons, in particular, weighing reasons against each other. In other words, if the separateness of persons is just the claim that reasons should not be aggregated across different people (Taurek, 1977), then the separateness of persons cannot explain why claims cannot be weighed across people, at pain of circularity.

For the theorist who rejects all interpersonal aggregation, the above is not a problem, as that rejection is just a bedrock commitment of their moral theory. However, I follow Broome here in taking claims to be a subset of reasons, distinct from reasons that can be traded off against each other and rights-based reasons. So, the move to reject interpersonal aggregation *tout court* is not available. In other words, the separateness of persons is supposed to explain the structure of claim-based reasons, rather than asserting that all reasons have a certain structure.^7^

Of course, one might adopt Broome’s account of fairness but reject his commitment to the interpersonal aggregation of reasons that are neither claims nor rights.^8^ However, an explanatory puzzle still remains. Fairness is, according to Broome, a matter of respecting claims in proportion to their strength. Part of what it is to treat people equally is to treat each person as an equal locus of value, whose nature and activities in the world generate claims. And, doing that requires responding to each person’s claims on the basis of their strength. I do not respect Fanele’s equal standing as an agent who generates claims if I treat her equally strong claims as less important than Gertrude’s. Nor do I treat Gertrude fairly, although she may not mind, as she benefits from this unfairness. And so, Broome’s account of fairness requires that the decision-maker is able to compare the strength of claims across people, at least in principle, and to allocate a good in proportion to the strength of the claim. Say, for example, that there is a dose of medicine that will alleviate Ezra’s debilitating migraine or Ferdowsi’s painful tension headache. The decision-maker needs to compare each of their claims to find a fair procedure to distribute the medicine that will best respect all of their claims. But, this is a similar structure of deliberation that one finds in a case where the decision-maker makes interpersonal tradeoffs to decide how to allocate a good by comparing the benefits and burdens to different people across different states of the world.

Here we can draw on recent work by Jessica Fischer (forthcoming) to further elucidate the separateness of persons objection in a way that distinguishes between interpersonal aggregation and the interpersonal comparison of claims. Fischer argues for a new account of the separateness of persons, understanding it as a complaint about the method by which a theory or an agent determines the right action. Theories or agents that violate the separateness of persons to do because they determine the right action *cross-personally*: they start by looking at the value that will be produced across different states of affairs. Theories or agents that do not violate the separateness of persons start with the individual, as well as special properties of that individual, as bearers of deontic assessment.^9^ But, starting with the individual is compatible with some of the properties of that individual being relational properties, such as having a stronger claim than someone else. And so, this view of the separateness of persons can help to flesh out why respecting claims in such a way as to respect the separateness of persons does not allow for the interpersonal aggregation of claims.

However, this raises the further question of what allocation procedures do respect claims, if interpersonal aggregation is off the table. This question will be addressed in Sect. 2.2 below, setting up the final piece needed for the argument against the use of decision thresholds in algorithmic decision-making.

### How can goods be allocated fairly?

The fair allocation of goods is easy when the claims to the good do not exceed the amount of the good. But, questions of fairness arise in conditions of scarcity. How should a scarce good be allocated in a fair manner, to respect the strength of people’s claims?

It is clear what Broome’s account requires in the case of divisible goods. A divisible good ought to be split between those people with claims to the good in proportion to the strength of their claims. If two friends are catching up over lunch, and one orders a meal twice as expensive as the other, then the person with more expensive habits ought to pay two-thirds of the bill, with the other friend paying the remaining one-third.

The harder question, however, is the question of what allocation procedure can respect claims in proportion to their strength when the good is scarce and is practically indivisible, i.e., either the good cannot be divided, or ought not be divided because the cost of doing so outweighs the benefits (Sher, 1980; Broome, 1984; Broome, 1990–1991). Say our friends above live in a culture where it is socially impermissible to split the bill. Which of the two ought to pay the bill? It seems as if any allocation procedure that allocates indivisible goods cannot avoid violating the separateness of persons, and thus is doomed to be unfair. That is because the people who lose out will not have their claims satisfied, whereas the person who benefits will. And fairness, recall, is a matter of the comparative satisfaction of claims, rather than their absolute satisfaction (Broome, 1990–1991; Piller, 2017). So, it may seem as if the fairest course of action is to allocate an indivisible, scarce good to no one.

In response, Broome and others in the literature have argued that lotteries best balance considerations of fairness and of the satisfaction of claims, when both are important (Voorhoeve & Fleurbaey, 2012; Piller, 2017).^10^ That is because the fact that someone had a chance at the invisible good matters morally for fairness. A chance at the good provides a “partial equality of satisfaction,” even though most of the individuals with a claim will lose out on getting the good (Broome, 1990–1991: 97). And, this partial equality of satisfaction is achieved by setting someone’s chance in the lottery equal to the strength of their claim. In other words, it is compatible with fairness if a party with a weaker claim to the good gets the good, as long as they did not have a disproportionate chance at the good.

The moral importance of chances for the fair distribution of indivisible goods explains why lotteries are often the fairest allocation method. The result of a lottery is that one or more people with a claim are allocated the good. However, lotteries distribute an *ex ante* chance at the good to all relevant individuals with a claim. There are important interpretative and normative questions about why, exactly, a fair distribution of chances achieves “partial satisfaction.” A common reading of Broome is that the fair distribution of chances achieves some *distributive* fairness (Spiekermann, 2022). Distributive views of the fairness of lotteries argue that lotteries are fair in virtue of an equal (or proportional) distribution of a benefit (Henning, 2015). In other words, a lottery is fair because it fairly distributes a benefit that people have reason to want - a chance at the good - even if the good itself cannot be distributed in such a way as to satisfy everyone’s claims to it. Of course, it cannot just be that the allocation procedure distributes *something* that participants have reason to want. If that were the case, then an allocation procedure that gave individuals with equal claims unequal chances at a good, but distributed a lollipop alongside the good, would be fairer than one that did not distribute the lollipop. The allocation procedure needs to in some way mitigate the *ex post* inequality in the distribution of the scarce good (Wasserman, 1996). Critics of distributional defenses of the fairness of lotteries argue that allocation procedures cannot do so, and that chances are not valuable in and of themselves (Hare, 2012; Henning, 2015; Lazenby, 2014). I will return to distributional views in Sect. 4.

There are two other defenses of why lotteries are fair that fit well with Broome’s argument for the fairness of weighted lotteries. Piller (2017) and Holm (2023) read Broome as holding a *procedural* view of the fairness of lotteries.^11^ Such views hold that lotteries are fair in virtue of treating people equally, not in virtue of distributing something people have reason to want. Two major questions for procedural views of lotteries are why a lottery treats people equally, and why they do so better than alternative allocation procedures. Holm (2023) takes lotteries to treat people equally because they do not override anyone’s claims by, for example, interpersonally aggregating them. We can use Fischer’s account of the separateness of persons to flesh this out further. Weighted lotteries respect the separateness of persons because they start from each individual and the strength of their claims as the fundamental bearer of deontic assessment, which are represented in the lottery by the different weights. Finally, one might give an *expressive* defense of the fairness of lotteries (Wasserman, 1996). This view is an alternative account of how one might cash out what it is to *respect* claims in proportion to their strength. Respecting claims is a matter of choosing a procedure that expresses certain attitudes, namely, the attitude that no one should be favored over others in cases where people have equal claims.

I tend to favor a procedural account of the fairness of lotteries, as it fits with Broome’s grounding of the concept of fairness in considerations about the separateness of persons. However, which account one adopts does not matter for my argumentative purposes. What is more important is the *form* that fair lotteries ought to take, according to Broome. Because Broome’s account of fairness states that claims should be satisfied in proportion to their strength, the weights of a lottery should be proportional the strength of each type of person’s claims. For example, if Hannes has roughly twice as strong a claim to life saving medicine as Juan, then Hannes should have roughly twice as great a chance as Juan to win the medicine lottery. Note that the use of a weighted lottery does not commit Broome to the view there is a fine-grained fact of the matter about the cardinal difference between the strength of claims (Piller, 2017). Here, we can import views from the philosophy of probability or utility theory to further spell out an account of weighted lotteries. Just as some have argued for imprecise credences, for example, we might argue for imprecise weights (Bradley, 2009; Keynes, 1921; Jeffrey, 1983; Joyce, 2005). Imprecise views seem to avoid the use of a sharp decision threshold to separate people with different features into different strengths of claims, which would help to avoid giving people with the same strength of claims different chances.^12^ In addition, a Broomean account of fairness does not have to hold that, for all types of claims, there are accurate and precise methods to measure the strengths of claims, contra Kirkpatrick and Eastwood (2015) (Piller, 2017). More generally, there may be many procedures for measuring the strengths of claims and attendant procedures for constructing a lottery or set of lotteries out of those strengths. The core arguments of the paper do not hinge on a view of the strength of claims.

Finally, it is important to note that, for Broome, reasons of fairness are *pro tanto* reasons. That a weighted lottery is a fairer allocation procedure than, say, distributing the good to the person with the greatest claim is a reason to use a weighted lottery. However, other reasons bear on which allocation procedure a decision-maker ought to use. As Broome discusses, the satisfaction of claims is also morally important (Broome, 1990–1991; Piller, 2017). If everyone has equally strong claims, then one fair allocation procedure is to allocate the scarce good to no one; but, such a procedure does very poorly at satisfying claims. For example, there may be reasons of comparative efficiency or overall welfare to avoid using a weighted lottery, and these reasons may outweigh reasons of fairness. In many contexts, it is plausible that reasons of fairness are outweighed by other reasons. In the example of the dangerous mission described in Sect. 2.1, for example, there are reasons of overall welfare to send the most skilled person that plausibly outweigh reasons of fairness. But, reasons of fairness will not always be outweighed by other types of reasons. Contrast the example of the dangerous mission with the allocation of places at kindergartens in a city. In such a case, reasons of fairness seem weightier than reasons of comparative efficiency or overall welfare.

This section developed a Broomean account of fairness as respecting claims in proportion to their strength, and motivated that weighted lotteries are a fair allocation method. The next section will use this Broomean account of fairness to defend three claims about algorithmic decision-making. The first claim is that the pervasive practice of using decision thresholds for algorithmic decision-making is sometimes unfair. The second claim is that weighted lotteries are a fairer way to do so. The third claim is that this rationalizes the use of methods in machine learning that build in diversity or multiplicity, such as ensemble learning or multi-task learning.

## Fairness and decision thresholds

The allocation of goods to which people have claims is increasingly automated. Algorithms are used to decide who gets a job, access to a loan, organs, and so on. I will focus on algorithmic decision-making systems trained to output a prediction or classification in a specific domain, rather than systems developed to accomplish more domain general tasks, such as generating linguistic content. Much of the literature on this topic has focused on moral questions raised by such systems themselves, such as questions about predictive fairness (Zimmermann & Lee-Stronach, 2022; Long, 2021; Hedden, 2021; Eva, 2022) or explainability (Vredenburgh, 2022; Babic & Cohen, 2023). However, more attention is needed on moral questions about how algorithmic predictions or classifications are converted into decisions, as well as how algorithmic systems impact the broader decision ecosystem (Creel & Hellman, 2022; Jain et al., 2023; Toups et al., 2023). Here I will focus on a feature of algorithmic allocation that has been relatively overlooked in philosophical discussions: the use of a decision threshold to convert a prediction to a decision.^13^

In computer science, the arbitrariness of decision thresholds has been argued to be one source of unfairness. If a decision-maker places the cutoff for a mortgage at a FICO score of 620, there will be some individuals that receive a score of 619 that are just as creditworthy as those that receive a score of 621. More generally, a single deterministic boundary for a classifier will lead to individuals just on either side of the boundary receiving different outcomes, which may strike one as unfair (Grgić-Hlača et al., 2017). This problem is a serious one, which I will return to in Sect. 4. Here, though, I will raise a different fairness charge against decision thresholds: that the use of any decision threshold is unfair, unless everyone above it has the same strength of claim, and everyone below it has no claim.

In what follows, I will draw on examples of goods to which individuals sometimes have claims. Readers, however, may find it implausible that individuals have claims to tokens of a good, such as a particular job, or to the general type of good, such as any job. Here, I will follow Broome in addressing this skepticism by distinguishing between claims and rights, when the latter are understood as side-constraints. Rights also generate directed duties, or duties owed to the particular individual who is the rights holder. But, rights cannot be overridden by other reasons. Claims, by contrast, can be overridden by other reasons (see Sect. 2.2). This distinction between claims and rights may help to soften the intuition that individuals do not have claims to goods such as loans or jobs. We can accept that it is implausible that individuals have a right to a token job, but maintain that, say, their qualifications generate a claim to that job. Selection criteria, for example, do not only serve an informational function to attract applicants that the employer most wants to hire. These criteria are also a public commitment to hire someone who fulfills those criteria, which grounds applicants’ claims in conjunction with the fact that they satisfy the criteria. As I will discuss in Sect. 4.3, claims are often partly grounded by institutional or organizational facts. Furthermore, in some cases, an individual may have a claim to a particular job or a particular loan. If the candidate is not only qualified, in light of the public selection criteria, but there is only one job in her small rural town that she is qualified for, and it is too costly for her to move to an area with more jobs, then her need partly grounds the claim as well. Finally, one need not be committed to the view that people ever have a claim to token goods of some types, such as jobs or loans. The arguments below go through for examples such as medical interventions as well. Furthermore, many of the arguments of this and the next section assume that individuals have a claim to *some instance of the good or opportunity or other*, an assumption that was defended in Sect. 2.1.^14^

Algorithmic allocation often proceeds via a two step procedure. The algorithmic systems targeted in this paper are, first and foremost, epistemic tools: they reduce uncertainty (Beigang, 2022; Hellman, 2020). Some algorithms aim to classify individuals as belonging to some group of interest, while others aim to predict facts about individuals. An algorithm may classify credit seekers into risk buckets, for example, which sort individuals based on how likely they are to pay back their loan on time. Or, an algorithm may predict an individual’s tenure on a job, as an input to a hiring process. Most predictions and classifications are either continuous or sort individuals into one of multiple different groups. Decisions, however, are usually binary. How should decision-makers go from a continuous prediction, for example, to a binary decision?

One option is to give the algorithmic output to a human decision-maker, who is then responsible for taking the output and making a decision. In a medical context, for example, a doctor might be given the information that an algorithmic diagnostic system predicted that a patient is 65% likely to have breast cancer based on their mammogram. It is then up to the doctor to combine this probabilistic information with their knowledge to come to a decision about further medical interventions. Another option is to automate not only the prediction but also the transition from the prediction to a decision. *Decision thresholds* are often used in algorithmic decision-making to automate the transition between a prediction into a decision. A decision threshold is a function from a prediction or classification to a decision. It specifies a cutoff above which the decision is yes and below which a decision is no. For example, imagine an algorithm that predicts an individual’s tenure on a job. The company may decide that they only want to hire an individual with a minimum predicted tenure of two years. So, they would consider everyone with a predicted tenure of two years or above for the job, and discard the others.

Of course, decision thresholds could be used outside of the context of algorithmic decision-making, and sometimes are. The arguments of this section apply to these uses of decision thresholds as well. But, we should expect to see a greater use of decision thresholds in algorithmic decision-making. Outputs of algorithmic systems are usually more fine-grained than human judgments and made on a single scale across a class of judgments. A FICO score, for example, is a discrete scale between 300 and 850 to measure consumer creditworthiness. In addition, the outputs are more transparent than the judgments of human decision-makers, who may lie about their beliefs. Transparent, fine-grained judgments on a single scale enable *consistent*, *efficient* decision-making, both of which are often seen as desirable. The use of a single decision threshold across candidates is usually taken to be necessary for fair decision-making, as it embodies a desirable consistency, both in theory but also in organizational practice (van den Broek et al., 2020; Mayson, 2019). In addition, a decision threshold further automates decision-making, potentially creating efficiency and thereby economic or welfare gains as well (Fourcade and Burrell, 2021). Furthermore, one could expect automated transitions from predictions to decisions to be more common in societies that view automated decision-making as more accurate, fair, and objective than human decision-making (Fourcade and Burrell, 2021; Porter, 1996). So, it is not surprising that we have seen the proliferation of decision thresholds alongside algorithmic prediction systems, more so than in cases of human decision-making.

The Broomean theory of fairness developed in Sect. 2 provides us with two necessary conditions for a decision threshold to be fair. The first condition is that the decision threshold cannot give some individuals with a claim to the good no chance at the good. The second condition is that decision thresholds must allocate chances at the good in proportion to the strength of claims. The first condition, of course, is entailed by the second: if chances at goods are allocated in proportion to the strength of claims, then individuals with a certain strength claim to the good will have a non-zero chance at the good. But, dialectically, it is helpful to separate them out; as we will see in Sect. 4, there are distinct arguments for the first and second necessary conditions, grounded in different components of the concept of fairness.

Violations of the first condition generate a fairness based complaint from those below the threshold who have a claim to the good. It is unfair, per the first condition, if individuals never had a chance to be allocated a good to which they have a claim. And, some decision thresholds deny people with claims a chance at that good. So, there is a *pro tanto* reason not to allocate goods using a decision threshold, when and because doing so would give those with some claim to the good no chance at the good.

Violations of the second condition generate a fairness based complaint from those above and below the threshold. In addition to the previous complaint, individuals above the threshold have a complaint that their chance at the good was not in proportion to the strength of their claim. That complaint arises because a decision threshold is binary: those above the threshold receive the good, and those below the threshold are denied it. Some thresholds, for example, will place individuals with different strengths of claims above the threshold. In such cases, the use of a decision threshold to allocate the good is unfair.

The discussion of the second condition that targets those with claims above the threshold may seem like a mere philosopher’s curiosity. After all, everyone above the threshold was allocated the good. Why would individuals with a greater claim than others have a complaint about chances, given that all of the individuals were allocated the good?

This objection, however, implicitly draws on an *ex post* account of fair allocation. According to *ex post* views, the fairness of an allocation is determined by the results from the allocation procedure, not by individual’s chances before the procedure is run (Hare, 2012; Lazenby, 2014).^15^ But, the arguments of this paper assume Broome’s *ex ante* account of fair allocation. Of course, one may not be committed to either an *ex ante* or an *ex post* view, but may still find the discussion of the second condition above implausible. Here, I would hazard that the argument’s plausibility increases on either a procedural view or an expressive view of the value of lotteries. Allocative procedures have an expressive function: they communicate the decision-makers’ understanding of people’s claims and respect for those claims (Piller, 2017; Spiekermann, 2022; Wasserman, 1996). Individuals with stronger claims may reject a decision threshold that gives them an equal chance to someone with weaker claims on expressive grounds, as the allocation procedure fails to communicate an equal respect for their claims.

Not all decision thresholds, of course, will generate fairness based complaints, as some decision thresholds give everyone with a claim to the good a chance at the good in proportion to the strength of their claim. One example is so-called adequacy-screening lotteries (Scanlon, 2018; Hussain, 2020). A threshold is used to separate those with claims from those with no claims, and everyone above the threshold has the same strength of claim. This structure of claims can arise when individuals have a conditional entitlement to a good. Suppose, for example, that individuals have an entitlement to a university education, conditional on fulfilling certain educational requirements. A threshold may be used to identify those with such a claim and an evenly weighted lottery used to allocate students to universities.

Decision thresholds are sometimes unfair because they fail to respect claims in proportion to their strength. But, as was argued in Sect. 2, weighted lotteries do respect claims in proportion to their strength. So, there is a *pro tanto* reason of fairness to allocate goods by lottery, rather than by decision thresholds. Of course, just because a weighted lottery is superior to decision thresholds on grounds of fairness, this does not entail that a weighted lottery is superior to all methods of random choice. Section 5 returns to this objection, arguing more conclusively in favor of weighted lotteries.

The two necessary conditions for fairness above can also provide an alternative grounds to research programs in computer science that seek more pluralistic decision-making by, for example, using ensemble learning or multi-task learning for fairer allocation. Let’s take an example of ensemble learning, in which multiple models are developed, rather than a single model (differences between learning algorithms is irrelevant for our purposes here). Each model might use different features or evaluation criteria, or be trained to achieve different objectives, or both (Jain et al., 2023). Sometimes, model predictions from ensembles are aggregated into a single prediction. A different approach, however, is to randomly select a classifier, and then use it to make a prediction (Grgić-Hlača et al., 2017). This approach for fair decision-making helps to satisfy the first necessary condition above. As long as everyone with a claim to the good would receive a positive classification on at least one of the classifiers, then random selection of a classifier gives each person some chance at the good (even though the chance is unlikely to be proportional to the strength of their claim).^16^

This section argued for two main claims. The first claim is that decision thresholds are unfair, because they do not allocate chances to the good in proportion to the strength of claims to the good. The second claim is that there is *pro tanto* reason to use weighted lotteries instead, where the weights are in proportion to the strength of claims, as such lotteries are fair. In addition, it sketched how these arguments support approaches to the development of algorithmic systems such as ensemble or multi-task learning. In the final two sections of the paper, I will consider objections to both of those claims in turn.

## Tie-breaking versus proportional satisfaction: in defense of a Broomean account of fairness

The arguments for the unfairness of decision thresholds hinge on a particular commitment of Broome’s account of fairness, that claims ought to be respected in proportion to their strength. But, the standard position in the literature is that lotteries ought only be used to break ties between equally strong claims. Below, I will argue against the standard position. Considering this objection also enables the further development of the Broomean case for the proportional satisfaction of claims by considering features of algorithmic decision-making that make a Broomean view especially compelling. I will first argue that equality demands that people have a chance to develop and pursue their conception of the good, but that this is stymied by the prevalence of interconnected data ecosystems marked by algorithmic monoculture at scale. However, any view of fairness must balance the importance of equality with the importance of satisfying institutionally-generated claims. I then argue that an account of fairness in terms of the proportional satisfaction of claims does so better than tie-breaking accounts, and that weighted lotteries best balance claims against using arbitrary or incorrect decision criteria with claims that arise from stable, institutionally-generated expectations.

### Should the strongest claim win out?

Broome’s commitment that claims ought to be respected in proportion to their strength is a minority position in the literature.^17^ Most theorists working on fairness and lotteries maintain that a good ought to be allocated to the individual with the strongest claim (Sher, 1980; Hooker, 2005; Stone, 2008). In many cases, there will be a tie among multiple individuals with the strongest claims to the good. Lotteries, this majority position goes, are only justified to break such ties between equally strong claims.

Sometimes it is taken as a conceptual truth that a person with the strongest claim ought to be allocated the good. Here is Sher (1980: 213):

It is part of our concept of strongest claims to goods, however, that when someone has such a claim, no one else is entitled to enjoy or dispose of the relevant good as he alone prefers. If n has the strongest claim to G, then any other person who either arrogates G to himself or delegates it to another on the basis of preference different from n’s is ipso facto infringing on n’s rightful claim to it.

According to this view, the Broomean account of fairness utilized in this paper is not, in fact, formulated in terms of claims, as it is part of the concept of a claim that the person with the strongest claim must be allocated the good.

Alternatively, one might ground the claim that lotteries should only be used to break ties in a general claim about how reasons work.^18^ In order to guide action, one might argue, reasons must be able to combine so as to determine an all things considered best or sufficiently good action. If one cannot aggregate reasons in order to determine the best course of action, then reasons cannot perform their function of guiding action. So, one ought to reject an account of reasons on which they do not aggregate.

Finally, one might argue by intuition. Here is a case by Hooker (2005: 349):

Suppose your claim on the medicine comes from the fact that you need it to save your life, and my claim on it comes from the fact that I need it to save my little finger. Suppose an average life is something like a thousand times more important than a little finger. So should the matter of who gets the medicine be decided by a lottery in which you have a 999/1000 chance of winning and I have a 1/1000 chance? Given that your claim is so much stronger than mine, how could it be right to take any risk that I rather than you might end up with the good? Letting the stronger claim win seems completely fair.

This case is intended to draw out the intuition that the person with the strongest claim should be allocated the good. It is not the cleanest case, as the benefit to be distributed is different for each party (Piller, 2017). It also opens itself up to the objection that the claim one has to be allocated medicine to save one’s little finger is *silenced* in a context of reasons such as saving someone’s life (Nagel, 1979).^19^ Still, many have the intuition that fairness requires that a person with the strongest claim be allocated the good.

Broome obviously does not take it to be a conceptual truth about claims that a claims is infringed if the good is allocated to someone else with a weaker claim. And, he denies that reasons of fairness can be aggregated or weighed against each other; he thus denies the general claim about reasons. But, Broome makes space in his moral theory for the aggregation of claims. Claims, according to Broome, ought to be satisfied, as well as respected proportionally. Claims can be aggregated in order to determine the action that satisfies the most claims (Broome 1990–1991; Piller, 2017). Fairness, however, does not allow for the aggregation of claims. Finally, Broome would not agree that it is “completely fair” to let the strongest claim win.

The debate between Broomeans and their critics is advanced enough that a knockdown argument to move critics is unlikely. But, Broome’s position is not as implausible as critics take it to be, especially in certain contexts of institutional decision-making. Below, I make the case for a Broomean concept of fairness in institutions that rely on algorithmic decision-making, drawing out general features that will be present in some non-algorithmic contexts as well.

### Equality, algorithmic monocultures, and not having a chance

What morally weighty complaint does an individual have if goods are allocated according to the principle that someone with the strongest claim should always win out? Individuals have a weighty moral complaint if, as Broome (1990–1991: 98) says, it was “never on the cards” that they would win. Spiekermann (2022) picks up on this thought to argue that lotteries are fairer than other allocation procedures because lotteries give each individual, winners and losers, a reason in favor of the lottery that they can accept, namely, that each person had an objective chance at the good. While I am sympathetic to Spiekermann’s argument, I am interested in *why* someone has a complaint if winning was never objectively on the cards. Below, I will argue for one answer as to why, and then use that answer in Sect. 4.3 to motivate weighted lotteries.

First, though, it is important to appreciate the strength of the complaint someone has if winning was never on the cards. The strength of this complaint can be drawn out if you place yourself in the situation of someone who never had a chance at a good to which they have a claim. Imagine that you live in a society where many people suffer from a kidney disease that is not life-threatening and whose severity ranges from mild to severe. You would greatly benefit from a kidney transplant, as you have a moderate kidney illness that significantly impacts your wellbeing. But, your government has built very little infrastructure for kidney transplants, nor have they invested in the development of new technologies. So, kidneys are scarce. Furthermore, the use of the decision criteria that prioritizes severe illness is highly standardized, i.e., the same criteria are used by all decision-makers. So, you will never be in a decision context in which you have one of the strongest claims to a kidney. Thus, you know that you will never have a chance at a new kidney. Finally, this chance is not merely an epistemic chance: it is not as if you had a chance but were not aware of it. Instead, you lack a real chance at the good. By “real chance,” I mean an objective chance, as opposed to a mere subjective credence that one had a shot at the good (Spiekermann, 2022). When you place yourself in the position of an individual who has no chance at an important good, it becomes, I hazard, more intuitively compelling that such individuals have a weighty moral complaint. And, Broome’s emphasis on the separateness of persons directs us to do just this: start from the individual and consider her claims, rather than comparing the amount of value produced across various states of affairs.

Before moving on to the defense of weighted lotteries as best balancing this complaint with others, it is important to appreciate how often this complaint will arise in societies with pervasive algorithmic decision-making. Various features of data collection, data sharing, and the training and deployment of AI systems will lead to so-called algorithmic monocultures. Algorithmic monocultures occur when decision-makers rely on the same model or when different models produce similar outputs (Bommasani et al., 2022; Creel & Hellman, 2022; Kleinberg & Raghavan, 2021; Toups et al., 2023). When there is an algorithmic monoculture, the same set of people are denied a good or opportunity across different decision-makers, either due to errors or because standardized criteria rule out those people. Algorithmic monocultures can occur because of the data. Model builders may use the same datasets because using less than all the available data would reduce model performance, in the case of Large Language Models trained on as much text data scraped from the internet as possible. Or, they may use all the available data because it is expensive to create the dataset. For example, a reason that so many data scientists use ImageNet is because it is a free image database that contains over 14 million labeled images. Algorithmic monocultures can also occur because different decision-makers purchase the same model from a company (Jain et al., 2023). If most companies purchase models from HireVue for recruitment, for example, then a job seeker will face the same decision criteria even though they are applying to different companies. Furthermore, because of data sharing across different decisions, we should expect clusters of disadvantage to accrue to types of people: if someone has a low credit score, she may be disqualified not only from loans, but also from jobs that use credit reports as a factor in hiring.^20^

Even if we do live in a society where algorithmic monocultures thrive, the proponent of tie breaking for equal claims will object that algorithmic monoculture itself is not a problem, as long as the good or opportunity always goes to the person with the strongest claim. They might object that those with the strongest claim could reject a principle that gives those with weaker claims some chance, and that those with weaker claims ought to accept distributional policies that exclusively favor those with the strongest claims. After all, had they been someone with the strongest claim, they too would have rejected any principle that gives those with weaker claims some chance. So, those with weaker claims who have no chance do not have a weighty moral complaint, as they ought to accept an allocation procedure that gives them no chance.

One way to respond to this objection is to push back against the purported obviousness of the judgment that those with weaker claims who never had a chance should accept an allocation procedure that gives them no chance. That judgment is, I hazard, strongest in one-off cases. Proponents of this view, such as Hooker (2005), often argue from a judgment about the fairness of a one-off decision, in which the good can be allocated to someone with a very strong claim or someone with a very weak claim. That judgment, however, can shift when one considers an individual with a weaker claim to some good facing a *series* of decision contexts in which they have no chance at the good in question, as in the kidney example above. These dynamic considerations bring out the strength of the complaint that an individual with some claim had no chance at the good.^21^

If that were the only response that could be given, we would be left with dueling intuitions. However, we can also explain why individuals have a morally weighty complaint if they have no chance within an institution whose rules partly or fully determine claims to a good. This explanation is rooted in the thought that these institutional rules are *arbitrary*. They are arbitrary in three senses. The first sense is that a set of institutional rules or principles is arbitrary from among the permissible set from behind the veil of ignorance, so to speak. There is a large set of permissible, equally good principles of justice that a society could choose to realize in its basic institutions, in order to satisfy values like fairness. A political community, for example, may choose between an economy organized as a property owning democracy or a market socialist economy, and private property entitlements will differ between these two economies. However, there will be a smaller set of permissible, equally good principles of justice that any given society can choose to realize in its basic institutions, as there are moral constraints on the choice of principles. One such important constraint is legitimacy, or how shared political power can be exercised to realize certain values (Schouten, 2024). Legitimacy may rule out certain principles, or may rule out certain actions by the state to achieve certain principles. But, social and cultural facts will also determine which principles of justice best realize the background values in a certain society (Schouten, 2024). And so, the second sense in which rules or principles can be arbitrary is that there are multiple, competing sets of permissible principles, holding fixed certain facts about that society that determine which principles best realize background values. The third sense in which institutional principles or rules are arbitrary is that the actual rules that front line decision-makers use are also arbitrary, as they are one of a number of permissible ways of operationalizing fuzzy institutional policies and values (Nguyen, 2020; Passi & Barocas, 2019). Front-line bureaucrats, for example, infamously face the problem of how to categorize individuals in order to determine whether certain rules apply to them or not (Zacka, 2017).

What complaints do individuals have who lose out in the face of arbitrary rules? There are three complaints that I will argue have the same, underappreciated grounds. The first complaint is that although someone is qualified for the good because they have a strong claim, given the set of existing values and background institutional rules at play, an arbitrary way of operationalizing those rules denies them the good. Discussions in computer science of the arbitrariness of thresholds, alluded to above, are focused on this complaint, which tracks the third sense of arbitrariness. The second complaint is that someone would have had a strong claim under a different, equally permissible set of institutional rules that could have permissibly been instituted in a given society with a certain culture and history. A different, permissible way of classifying the severity of disease, for example, could lead to the individual in the kidney example above having a stronger claim to a kidney, and the rules would do just as well to permissibly distribute scarce health resources. So, an individual has a weighty complaint against having no chance at the good when the rules are arbitrary, which tracks the second sense of arbitrariness. This complaint has also been recognized in the literature on algorithmic allocation. Jain et al. (2023), for example, use Fishkin’s (2014) criticism of equal opportunity to argue that an ecosystem of decision-making algorithms ought to create chances at valuable goods and opportunities across people with different talents and skills, needs, or histories. Fishkin’s view is grounded in the value of opportunities for people: everyone should be able to access opportunities that are valuable to them and for them. So, according to Fishkin, we ought instead to promote what he calls opportunity pluralism, in which multiple types of opportunities receive social esteem, and there are multiple pathways to them, each of which favors different talents and dispositions.

In order to make sense of Fishkin’s revised account of equality of opportunity, and Jain et al. (2023) use of it to argue against algorithmic monocultures, we have to restrict his account to a complaint against the selection of permissible, equally good principles in the context of a given society. However, I take Fishkin’s starting point to lead us to a more radical view of the implications of the concept of equality for allocative fairness, one that starts with the value of equality, rather than the value of opportunities (Schaar, 1967). As Schaar (1967) notes, in any society, not all talents can be developed equally; even if there are multiple avenues to different opportunities, there cannot be a set of opportunities nor pathways large enough to reflect everyone’s talents, dispositions, and interests. But, this limitation limits the ability of any society to realize the fundamental equality of persons, understood as the ability of each person to formulate their own conception of the good and pursue their life plan, consonant with the ability of everyone else to do the same. And so, even those who face the third kind of arbitrariness, where there is no best and permissible set of opportunities and attendant criteria that can be instituted in their society, have a significant complaint. And that complaint is grounded in the equal claim each person has to pursue their life plans.

What kind of allocation procedure best recognizes the strength of this complaint while also recognizing the legitimate claims of people within a particular society? I will now defend the moral superiority of weighted lotteries over random tie-breaking for equally strong claims, from the perspective of fairness.

### In defense of weighted lotteries

Above, I defended the claim that people have a moral complaint if they have no chance at justice-relevant goods that are allocated by institutions that rely on arbitrary decision-making criteria. This is a complaint that becomes increasingly frequent in the actual world because of how algorithmic decision-making has been implemented in many societies. So, it is interesting theoretically, but also important practically, to figure out if there is an allocation procedure that can address this complaint without creating larger complaints from others under that alternative procedure.

To theorize about that question, I use a contractualist approach to the justification of allocation procedures. Contractualist views take morality to be a matter of principles that no one could reasonably reject. They are a natural complement to Broome’s theory of fairness because they are non-aggregative approaches to social risk imposition (Scanlon, 1998, 2013; Frick, 2015). Even if one is not a contractualist about morality, however, the contractualist framework offers helpful heuristics to systematize our moral reasoning about the justifiability of institutional arrangements in terms of what morally weighty complaints individuals have against different possible arrangements.

In Sect. 4.2, I argued that individuals have a weighty moral complaint if they have no chance at justice-relevant goods that are allocated by institutions that rely on arbitrary decision-making criteria. Is there an alternative principle that mitigates this complaint without creating weightier complaints? Two initially attractive alternative principles fail the contractualist test, as they create weightier complaints. The first principle I take to be a non-starter in the cases in which we are interested. That is the principle of an equal entitlement to the good. In the cases we have discussed, like medical resource allocation, hiring, or credit, a key background assumption is that the good is permissibly scarce; in other words, the resource and other costs of producing enough of the good to satisfy such an equal entitlement would create weightier complaints. Otherwise, if there are claims to the good, then the importance of satisfying claims creates a duty to produce more of the good, when it is not too costly.

Another principle is the policy of randomizing equally amongst everyone with claims. But, individuals with the strongest claims have a complaint against giving everyone an equal chance. Here, a further assumption is necessary to make this point: that institutions are using morally permissible criteria that have two further good-making features, namely, publicity and creating stable expectations about one’s claims and their strengths. Here I am drawing on a tradition in political philosophy according to which claims are partly, or largely, determined by institutional facts. A common liberal position, for example, is that natural endowments only have moral significance in virtue of background institutional facts (Rawls, 1972). Institutional facts, plus natural endowments, determine claims, in cases where the claim is grounded in the achievement of certain qualifications, or based on institutional promises. In addition, those institutional rules ought to have two further good-making features. They ought to be public, in order to satisfy considerations of legitimacy (Rawls, 1972), and to enable the agency of those qualifying for scarce goods within that institution (Vredenburgh, 2022). And, they ought to create stable expectations of who gets what over time. In other words, claims are “specified by the public rules of the scheme of social cooperation” (Rawls, 2001: 72). The moral weight of these claims is grounded in the promise between the individual and the institution: when individuals conform their behavior to institutional rules, they gain claims with a certain strength, in light of what the rules state their claims are. Individuals whose chances would be reduced by the randomizing procedure have a complaint that the procedure does not respect their claims to the good, which are backed by institutional promises.

This account of what determines claims - institutional rules, plus facts about how individuals satisfy those rules - helps to answer an objection to the argument of Sect. 3 that the use of weighted lotteries is fairer than the use of decision thresholds. To use a weighted lottery, there must be a method to sort people into types, where each type has a different strength of claims. However, one might worry that the properties of people that ground claims ought to be mapped onto a continuous scale: someone with a certain illness may have one year and one day to live, and another person one year and five days, for example. In order to sort people with such properties into types with a certain strength of claim, the decision-maker has to use a threshold. And so, the same objection that I made against the use of decision threshold can be leveraged against my claim that weighted lotteries are fairer than decision thresholds.

This objection, however, is more compelling if one thinks about claims as determined by natural facts. If claims were entirely determined by natural facts, it is more likely that the properties of people that ground claims can be represented on a continuous scale, creating the problem above.^22^ However, if claims are partly grounded in institutional criteria to allocate a good, then the facts that ground them are more likely to divide people into categories with sharp and arbitrary thresholds, avoiding the problem above. Of course, even if claims are partly grounded in institutional facts, this might not create sharp categories of people with different strengths of claims, as claims are also grounded in non-institutional facts. In that case, we can appeal to the importance of satisfying claims as an additional grounds of the goodness of weighted lotteries. Lotteries also help to ensure that at least some claims are satisfied, namely, the claims of the winners of the lottery. And so, a lottery which mistakenly miscategorizes the strength of some people’s claims might still best balance the good of having an efficient procedure for satisfying claims with the importance of giving each person a chance, and respecting claims in proportion to their strength.

However, it was exactly that arbitrariness that created the significant complaint when someone does not have a chance at the good. And so, arbitrariness arises to solve one problem, and create a certain good - stable, transparent expectations that ground claims - but it also creates a significant complaint on grounds of equality. We seem to be stuck. The equality of persons, which is one core component of the concept of fairness, is a ground on which someone can reject an allocation procedure that gives them no chance at the good, even though they have a claim. However, someone with a stronger claim can reject the principle that everyone is given an equal chance at the good, on the grounds that each ought to be given their due, and they are not given their due, as determined by institutionally-generated claims. The principle that those with claims ought to have a chance in proportional to the strength of their claim mitigates the complaint of those who would otherwise have no chance at the good and is tolerably costly to all, as the institutional entitlements of those with stronger claims are respected. Thus, the fairest allocation procedure balances these two complaints in the form of a weighted lottery. Those with stronger claims have a greater chance at the good, but those with weaker claims have some chance, which respects equality.

However, this defense of weighted lotteries seems to run into a highly counter-intuitive result. If there are grounds of equality to give everyone a chance, then it seems as if a weighted lottery should allocate people without any claims, as determined by the institutional rules, a small chance at the good. After all, they may have a very small claim in virtue of other facts, such as facts about their life plan. And, since there will be many more such people with extremely small claims than people with larger claims, one might worry that someone with an extremely small claim will end up getting the good in question, which reduces claim satisfaction and overall welfare. Let’s consider this worry in the context of jobs. Jobs are important for people to realize their life plans, and so people seem to have a small claim to a job for which they are unqualified. Someone without any musical training, for example, would have an extremely small claim to a job in an orchestra. If there are far more people who never had musical training but desperately want to play in a professional orchestra than there are trained musicians, then we will end up with too many failed orchestras.

There are a few responses that are open here. One response is that there are not reasons of equality to give *everyone* a chance at every good. A person without liver disease does not need a liver in order to pursue her life plans; or, someone does not need a particular job in order to have a fair and equal opportunity to develop and pursue her life plan. In such cases, people do not have an equality-based complaint if they have no chance at the good. Because there are not reasons of equality to give everyone a chance at the good, those with weaker claims will not win out too often. The second response is that, in other cases, considerations of claim satisfaction or overall wellbeing support the design of a weighted lottery where those with a very small claim are not given a chance at the good. And the third response is that, in some cases, this is a feature, not a bug: it is indeed not fair if those with a small claim had no chance at the good.

By taking the complaint that individuals would have if they have no chance as a good seriously, we arrive at a new argument for weighted lotteries, grounded in the need to balance complaints due to arbitrary criteria with complaints that arise when institutionally-generated claims are overridden. This tension will be particularly acute in societies with pervasive algorithmic decision-making, as algorithmic decision-making both creates algorithmic monocultures, but also creates more precise and transparent criteria. And, what this complaint reveals is not only an insight about the moral problems raised by algorithmic monocultures; it also reveals that proponents of a Broomean account of fairness have focused too much on Aristotle’s dictum that fairness is a matter of giving each their due. Equality is also important for fairness; but, Broomean theories often do not tell us what equality is, nor draw on the notion to support the fairness of weighted lotteries. This section fills in that gap in the literature.

The arguments above only target the objection that decision thresholds are fair, when and because they give the good to those with the strongest claims. It doesn’t yet settle the question of whether a lottery is the fairest procedure. In the final section, I will tackle the issue of allocation by lottery.

## Are lotteries required for fair allocation?

The claim that there is *pro tanto* reason to use weighted lotteries instead of a decision threshold faces a further challenge. Henning (2015), for example, has argued that there is no moral reason to prefer lotteries over other methods of allocation that give individuals some chance at the good, such as arbitrary picking. Responding to this challenge will bring out strengths of Broome’s account of fairness over competitors.

Let’s say that the objector grants the arguments of Sect. 4 and accepts that many allocation procedures are fair in virtue of distributing chances to the good in proportion to the strength of claims. However, they might push back against the claim that lotteries in particular are required for fair allocation, over other methods of allocating goods that distribute chances to the good. Henning (2015) argues against what he calls the Lottery Requirement, or views that claim that lotteries are superior to other allocation methods for reasons of distributive or procedural fairness. He targets three families of views that purport to establish the requirement: surrogate satisfaction views, procedural accounts, and ideal consent accounts. For reasons of space, I will focus on Henning’s argument against surrogate satisfaction views.

Surrogate satisfaction views take an equal chance at the good to be a benefit that is distinct from the good itself. And, the view goes, lotteries best distribute this additional benefit, which is valuable in cases where not everyone can get the good itself. In response, Henning grants that individuals have reason to want the good, and reason to want a chance at the good. But, Henning argues, a chance at the good is not valuable in and of itself; instead, it is only valuable because one may get the good. Otherwise, one is committed to implausible verdicts in cases like the following: A decision-maker is choosing whether to save you or another person, and cannot save both. They give you the choice between two procedures, A and B. Procedure A is a fair lottery. In Procedure B, the decision-maker saves whoever’s name is written on a piece of paper. You have some evidence that your name is more likely to be on the piece of paper. According to Henning, the correct verdict about the case is that the benefit of B outweighs the benefit of A, no matter how small the probability. But, if Procedure A distributed a benefit other than the good, you would need to know the probability that your name is written on the paper, to choose a procedure based on the expected benefit to you.

Henning concludes that none of the arguments in favor of the Lottery Requirement work. Its intuitive appeal can be explained away by the plausibility of nearby claims: that individuals have an interest in having some chance at a scarce good that would benefit them, or that procedural fairness requires that the decision is not influenced by problematic partiality (Kornhauser & Sager, 1998). But, Henning argues, all those claims establish is that individuals have an interest in the decision-maker choosing arbitrarily, or choosing for no particular reason. Lotteries are one instance of a procedure that results in an arbitrary choice. But, there are other procedures that would result in an arbitrary choice in certain contexts, such as choosing the person whose name begins with the letter closest to the letter ‘a’.

Someone who holds that goods should go to someone with the strongest claim is on the hook to rebut Henning’s criticisms. A Broomean, however, can grant that Henning’s argument succeeds in showing that the Lottery Requirement is false, as Henning’s arguments do not undermine a Broomean case for weighted lotteries. That is because Henning formulates the Lottery Requirement in terms of conflict cases, e.g., cases where individuals have *equal* claims to a good. But, the arguments do not extend to cases where individuals have differing strengths of claims. When individuals have differing strengths of claims, it does not just matter that individuals have some chance, according to Broome; the comparative chances matter. But, other methods of arbitrary choice, such as picking, do not distribute chances in proportion to the strengths of claims, except by a massive coincidence or an artificial set up.

This response also helps to respond to a different, but related, objection. The objection is that a lottery is not required to distribute goods for which there are conflicting claims because a so-called natural lottery has already been run. This objection comes up in the literature on partiality and rescue cases.^23^ Say that an individual faced two groups that needed rescue and could only save one group. One group has three individuals, and one group has seven. Some have defended the view that a weighted lottery ought to be run, where each individual has an equal 1/10 chance to be saved. But, the objection goes, running a lottery would be double counting, because each individual already had a 1/10 chance of ending up in each group (Henning, 2015).

However, this objection is only compelling because each individual’s chance reflects their equal claim.^24^ But, when claims different in strength, the chances will only mirror the strength of claims in artificial or rare cases. We can grant that all the individuals in the rescue case happened to choose to swim to one island or another, where they were then stranded, and that these chancey choices are describable as a lottery. But, say that some individuals had a stronger claim to be saved than others. What chancey process in the world would lead them to swim to an island such that the strength of their claims matched the chance that they would be saved on that island?

A further benefit of Broome’s view, then, is that it has the resources to argue against alternative accounts of random choosing, such as arbitrary picking. For Broome, a fair lottery is one in which the chances are proportional to the strength of the claims. And, an allocation mechanism must be designed so that the chances correctly reflect the strength of peoples’ claims. Artificial lotteries thus have an advantage over alternative randomizing procedures like arbitrary picking or natural lotteries, as the latter do not guarantee that the chances correctly reflect the strength of peoples’ claims.

## Conclusion

This paper argued against the use of decision thresholds, when and because they are unfair. Instead, weighted lotteries should be used, where the weights are proportional to the strengths of claims.

The arguments of the paper are especially important in a world in which AI partly determines the allocation of justice-relevant goods (Gabriel, 2022). Big data and AI have created conditions in which more individuals with claims to goods have no real chance at them. Algorithmic decision-making is often touted as fairer because it reduces randomness in decision-making (Kahneman et al., 2021; Sunstein, 2022). But, if the above arguments are correct, individuals have serious fairness complaints against algorithmic decision-making that reduces randomness such that they have no real chance at the good in question. And, there is good reason to think that algorithmic decision-making will tend to reduce randomness in such a way as to give those with some claim no chance, as was argued above. Such standardized decision-making raises serious complaints from those who have no chance at the good, even if those individuals are not mischaracterized, e.g., the decision-maker accurately detects the strength of their claim.^25^ Institutional decision-makers thus have reasons of fairness to be wary of using algorithmic systems to allocate goods.

## Footnotes

1 For another use of Broome’s account of fairness to theorize about algorithmic fairness, see Holm’s (2023) argument that fairness metrics express different accounts of what grounds a claim.

2 Broome 1990: 95 calls this the principle of “horizontal equity.”

3 Aristotle, 1998 III.13; quotation taken from Zimmermann & Lee-Stronach, 2022: 16.

4 See, for example, Meyer (2018), who argues for a right to credit on broadly these grounds.

5 As Broome (1990–1991: 92) discusses, the distinction between claims and overall benefit does not track deontological vs. consequentialist views of morality, as some historical utilitarian thinkers have held that an individual has a claim that others perform an act that will benefit them, although it would not produce the most good. Some contemporary welfarist prioritarian theorists of distributive justice also hold such an account of claims (see Adler, 2022).

6 See Rawls (1972), Nozick (1974), and Nagel (1979) for some canonical discussions of the separateness of persons.

7 There is an additional response here that I do not explore, as I do not find it to be particularly promising. One could read Broom as claiming that the separateness of persons is a metaphysical fact, and this metaphysical fact grounds the normative prohibition against interpersonal aggregation. However, it is left mysterious why it does so. And, the proponent of the separateness of persons objection is left with no compelling response to consequentialists that insist that interpersonal aggregation does respect the separateness of persons, as consequentialist theories determine the rightness of an action by combining the value that action produces across each individual person affected (Hirose, 2013). Here I agree with Fischer (forthcoming) that we are owed an explanation of why the separateness of persons rules out interpersonal aggregation.

8 Many non-consequentialists have taken the rejection of aggregation to be a morally implausible commitment, and instead defended more limited aggregation principles (Brink, 1993).

9 This view is also hinted at in Taurek (1977), discussing a case where a drug can be given to save the lives of a group or five people or to save the life of one person: “My concern for what happens to them is grounded chiefly in the realization that each of them is, as I would be in his place, terribly concerned about what happens to him. It is not my way to think of them as each having a certain objective value, determined however it is we determine the objective value of things, and then to make some estimate of the combined value of the five as against the one. If it were not for the fact that these objects were creatures much like me, for whom what happens to them is of great importance, I doubt that I would take much interest in their preservation … It is the loss *to this person* that I focus on.” (Taurek, 1977: 306–307).

10 To simplify the arguments of this paper, I will assume that decision-makers are in a state of certainty about what people’s claims are. However, uncertainty about what claims are relevant to a given decision, or who has a feature that grounds a claim of a certain strength, raises important questions. I would hazard that the use of weighted lotteries would satisfy more claims under either conditions of certainty or uncertainty than the other two alternatives considered in this paper (the use of a single decision threshold, or tie-breaking between the strongest claims). Take a case of uncertainty about people’s features, in which Wendy has five times as strong a claim as Xavier to life saving medicine, and Xavier has twice as strong a claim as Ying to that medicine. But, the decision-maker takes Xavier to have a 50% chance of having a feature that makes his claim twice as strong as Ying, and a 50% chance of having a feature that makes his claim equal to Ying’s. They decide to use a decision-procedure that is risk averse, classifying Xavier as having an equal claim to Ying. Let’s stipulate that Ying is below some given threshold for the good, and so Xavier is as well, and he has no chance at the good. If a weighted lottery is used instead, then Xavier has some chance at the good. This procedure is fairer than the use of the decision threshold, on the three accounts of the fairness of lotteries. The procedure is not perfectly fair on a distributional account of the fairness of lotteries: Xavier is given something he has reason to want, but he is not fully given what he is owed. On an expressive account of the fairness of lotteries, the weighted lottery may be perfectly fair, as it expresses the decision-maker’s beliefs about the strengths of people’s claims. We would need a more detailed procedural account to evaluate whether such a lottery is fair: the procedure does take each individual’s claim to ground what to do, respecting the separateness of persons, but whether it is completely fair will depend on whether one takes there to be independent facts about the strengths of claims, and whether a fair procedure requires a decision-maker to decide according to her best evidence about claims, or according to the actual strengths of people’s claims. Thanks to Reviewer 1 for raising this important point.

11 For further procedural defenses of the fairness of lotteries, see Stone (2008).

12 Thanks to Reviewer 1 for pushing me to discuss the implementation of a lottery and raising the problem of sharp thresholds against my own view.

13 There are scattered discussions within philosophy of moral questions related to decision thresholds. Long (2021) notes that where to set the threshold is a moral matter, as it determines the distribution of false positives and false negatives across people. Another example is Birch et al. (2022), who argue that the use of a single decision threshold for AI diagnostics is ethically problematic because it ignores the different risk attitudes of patients.

14 Thanks to Reviewer 2 for pushing me to defend the use of particular examples of goods to which agents have claims throughout the paper.

15 Or, one might hold a hybrid view, on which fairness in the outcome of the allocation procedure is lexically prior to the fair distribution of chances (Lazenby, 2014). Such a view allows facts about the distribution of chances to contribute to the fairness of an allocation, but still denies that a weighted lottery is fairer than a distribution of the good to the person with the strongest claim.

16 As Grgić-Hlača et al. (2017) say, random selection from a set of classifiers means that “there is a non-zero chance of both beneficial and non-beneficial outcomes.”

17 For another defense of the use of weighted lotteries for the fair allocation of claims, see Kornhauser and Sager (1998).

18 Piller (2017) attributes such an account to Hooker (2005). Since claims are reasons, and “we always ought to act in accordance with the strength of reasons” (Piller, 2017: 228), we ought to allocate the good to someone with the strongest claim. Thanks to Sebastian Köhler for pushing this point in conversation.

19 See also Piller (2017) for an argument that Broome’s account includes a measure of *ex post* fairness; thus, when claim strengths diverge greatly, it would be more unfair to hold a lottery. This argument addresses the objection that Broome’s view leads to implausible results when claim strengths differ greatly.

20 This is a similar point to Hellman’s (2023) argument that algorithms can compound injustice, but does not assume that there was an original injustice.

21 Arguments that focus on the distributional effects of lotteries over time often focus on considerations of fairness between groups. We could imagine that members of group A and group B both had equal claims to some scarce good, but decision-makers happened to choose arbitrarily in such a way that only members of group A received the good. The introduction of an objectively chancey lottery can, in the long run, go some way to ensuring that there is not an uneven distributional impact on one group (Wasserman, 1996). The argument here, by contrast, focuses on questions of individual fairness.

22 Thanks to Reviewer 1 for raising this objection.

23 Thanks to Louis-Philippe Hodgson for pressing this point.

24 A further objection argues that such cases of genuine real-world lotteries will be rare. Elster (1989), for example, assumes that mere randomness is what matters for fairness; thus, for Elster, the timing of the random process does not matter (see Spiekermann, 2022 for discussion). But, as Henning (2015) argues, one can grant that chancey processes are pervasive without granting that such chancey processes are lotteries. And, lotteries are important for fairness, not mere chance. However, as Henning notes, an argument is needed to explain why all chancey processes are not lotteries.

25 This point distinguishes the arguments of this section from Creel and Hellman (2022), who argue that arbitrary decisions are unfair when and because they are systematic, locking individuals out of important opportunities. And, they argue, algorithmic systems are more likely to be systemic in this morally troubling way.
